# Assessing mental health in Aboriginal youth

**DOI:** 10.1192/j.eurpsy.2022.375

**Published:** 2022-09-01

**Authors:** A. Janca, Z. Lyons

**Affiliations:** University of Western Australia, Division Of Psychiatry, Perth, Australia

**Keywords:** Assessment, culture, youth, Aboriginal mental health

## Abstract

**Introduction:**

The assessment of social and emotional wellbeing (SEWB) among Aboriginal people in Australia and elsewhere is complex and challenging task. A culturally appropriate tool for screening SEWB among Aboriginal adults known as the *Here and Now Aboriginal Assessment* (HANAA) has been developed and evaluated. The HANAA is based on exploring key domains of Aboriginal concept of SEWB and is based on a yarning process aimed to initiate a semi-structured interview that covers each domain. Over the last ten years the HANAA has been widely used by Aboriginal mental health service providers around Australia and elsewhere.

**Objectives:**

There have been multiple requests by service providers for a similar tool to be developed for young Aboriginal people. The aim of this study was to develop a youth version of the HANAA.

**Methods:**

A Working Group was established to guide the development of the youth HANAA. This work included discussion of assessment domains, prompt words and other adolescent specific considerations that were needed. The evlauation process was also discussed.

**Results:**

The adult version of HANAA was well accepted by participants. Reliability was good with kappa agreements between Aboriginal and non-Aboriginal interviewers ranging from 0.5 to 1.0. Agreement between interviewers and treating clinicians on ecommended course of action was good.

**Conclusions:**

Based on the previous field test results, it is expected that the youth HANAA will also be a culturally appropriate and useful tool which can be used by a range of service providers with differing levels of mental health training to assess SEWB among young Aboriginal people.

**Disclosure:**

No significant relationships.

